# Acute vs. chronic vs. intermittent hypoxia in breast Cancer: a review on its application in *in vitro* research

**DOI:** 10.1007/s11033-022-07802-6

**Published:** 2022-09-03

**Authors:** Qiuyu Liu, Victoria A.C. Palmgren, Erik HJ Danen, Sylvia E. Le Dévédec

**Affiliations:** Division of Drug Discovery and Safety, Leiden Academic Centre of Drug Research, Leiden University, Leiden, The Netherlands

**Keywords:** Breast Cancer, Acute Hypoxia, Chronic hypoxia, Intermittent hypoxia, Hallmarks of Cancer

## Abstract

Hypoxia has been linked to elevated instances of therapeutic resistance in breast cancer. The exposure of proliferating cancer cells to hypoxia has been shown to induce an aggressive phenotype conducive to invasion and metastasis. Regions of the primary tumors in the breast may be exposed to different types of hypoxia including acute, chronic or intermittent. Intermittent hypoxia (IH), also called cyclic hypoxia, is caused by exposure to cycles of hypoxia and reoxygenation (H-R cycles). Importantly, there is currently no consensus amongst the scientific community on the total duration of hypoxia, the oxygen level, and the possible presence of H-R cycles. In this review, we discuss current methods of hypoxia research, to explore how exposure regimes used in experiments are connected to signaling by different hypoxia inducible factors (HIFs) and to distinct cellular responses in the context of the hallmarks of cancer. We highlight discrepancies in the existing literature on hypoxia research within the field of breast cancer in particular and propose a clear definition of acute, chronic, and intermittent hypoxia based on HIF activation and cellular responses: (i) acute hypoxia is when the cells are exposed for no more than 24 h to an environment with 1% O_2_ or less; (ii) chronic hypoxia is when the cells are exposed for more than 48 h to an environment with 1% O_2_ or less and (iii) intermittent hypoxia is when the cells are exposed to at least two rounds of hypoxia (1% O_2_ or less) separated by at least one period of reoxygenation by exposure to normoxia (8.5% O_2_ or higher). Our review provides for the first time a guideline for definition of hypoxia related terms and a clear foundation for hypoxia related in vitro (breast) cancer research.

**Supplementary Information:**

The online version contains supplementary material available at 10.1007/s11033-022-07802-6.

## Introduction

Given the problem of therapy resistance in cancer treatment, the field of hypoxia research has grown in importance as a result of the crucial role hypoxia has in the therapeutic response. Tumors exposed to hypoxia have been shown to have a more aggressive phenotype characterized by increased metastasis and resistance to chemotherapy, radiation, and inducible cancer immunotherapy [1]. More specifically, hypoxia and genomic instability are correlated in breast cancer and genomic instability is associated with aggressiveness in several tumor types [2]. There is good evidence that intratumoral hypoxia is commonly found in breast cancer, and that breast cancer cells have elevated expression of hypoxia-inducible factors (HIFs) [3]. There are negative implications for patient survival, independent of clinical stage, prognostic parameters, histological grade, and lymph node status [4, 5]. Therefore, it is crucial to gather a stronger understanding of the impact of hypoxia to strategically circumvent its effects on breast cancer progression and therapy resistance.

Hypoxia can be caused by uncontrolled proliferation of cancer cells which distances them from the nearest source of oxygen [6]. The distancing from the nearest oxygen source results in a varying hypoxia profile from acute hypoxia to chronic hypoxia. Acute hypoxia is defined by short (from a few minutes to a few hours) exposure to low oxygen levels that can be reversed by regained blood flow, whereas chronic hypoxia is defined by long exposure to low oxygen levels. Hypoxia can also occur in a cycling, or intermittent phase which arises due to the shutdown of immature, dysfunctional structure of the new vasculature resulting in transient blood flow [6, 7]. Intermittent hypoxia and reoxygenation have been shown to have a stronger effect on promoting an invasive breast cancer phenotype than chronic hypoxia [8]. Interestingly, cells that are exposed to intermittent hypoxia preferentially undergo glycolysis despite the fluctuating availability of oxygen, which signifies that these cells acquire adaptability to the microenvironmental stresses that they may experience during metastasis [9]. The severity and duration of hypoxia defines the cell response and thus the subsequent signaling mechanisms initiated by the cancer cell [6]. Cancer cells that undergo both chronic and intermittent hypoxia display a phenotype that is characterized by higher reactive oxygen species (ROS) defense [10], and upregulation of genes involved in the metastatic spread[9] .

In this review, we summarize the current standards on how the scientific community uses hypoxia in in vitro breast cancer research. We review studies with respect to the duration of hypoxia, oxygen levels, and biological effects of acute, chronic or intermittent hypoxia. Not surprising but worrisome, we observe a large discrepancy amongst the studies with respect to the duration required to induce an acute hypoxic or chronic hypoxic phenotype. For instance, Jarman et al. referred to 24 h as acute hypoxia, whereas Han et al. used 24 h as chronic hypoxia [11, 12]. Bayer et al. classified acute hypoxia as less than 2 h of hypoxia exposure, whereas chronic hypoxia was anything longer than 2 h [13]. The inconsistent classification of hypoxia is based on experimental observations solely and largely disregards the multiple pathophysiological and pathogenetic processes involved [13]. This highlights the urgent need of understanding the literature and drawing conclusions on the most representative methods for conducting hypoxia research in the breast cancer field.

**Fig. 1:**
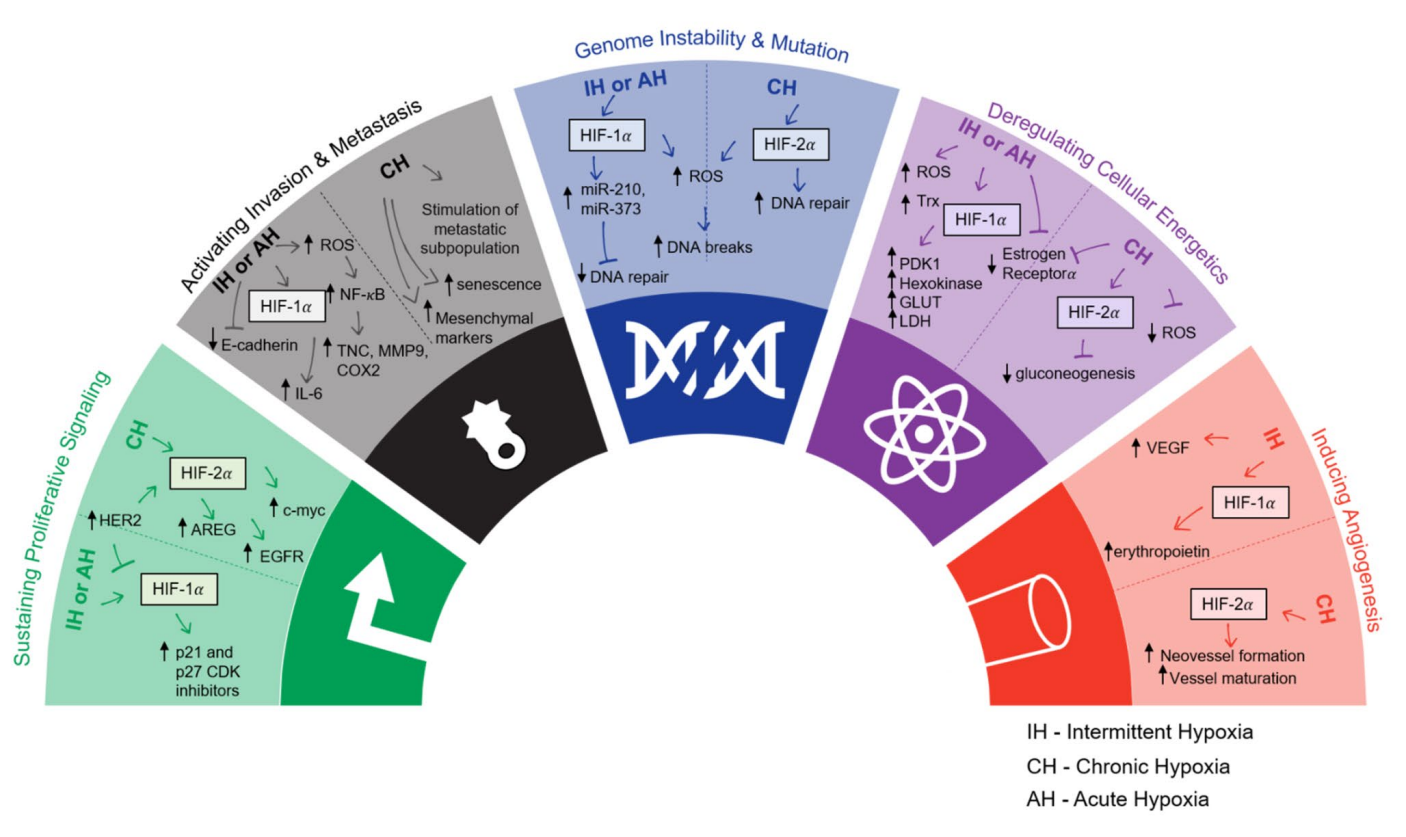
The role of acute/ chronic hypoxia (AH/ CH) and intermittent hypoxia (IH) in a selection of the hallmarks of cancer. Adapted from Hanahan and Weinberg 2011. References found in Table 1

Here, we discuss potential reasons for the major differences in the duration and biological effect reported for experimental acute, chronic and intermittent hypoxia. We classify our findings based on the hallmarks of cancer **(**Fig. 1**)**, detailed by Hanahan and Weinberg, in order to allow researchers to tailor the optimal experimental setup for hypoxia based on what they want to study [14]. This review also provides an overview of the different strategies being applied to hypoxia research within the field of breast cancer **(**Table 1**)**, and ultimately, we provide new guidelines for future research.

**Table 1:** Effects of Intermittent and Acute/ Chronic Hypoxia on In Vitro models of breast cancer

Reference	Cell Line/ Mouse Model	Conduction of Hypoxia	Intermittent Hypoxia (IH)	Effect of IH	Acute Hypoxia (AH) or Chronic Hypoxia (CH)	Effect of AH/ CH	Hallmark of Cancer [90]
[59] Alhawarat et al., 2019	MCF-7	AnaecroGen System	8 h (1% O_2_), three times per week	Increase in VEGF secretion Acquired higher chemoresistance Increased stemness properties	1% O_2_ for 72 h, once per week	Same results at effects of IH	Inducing angiogenesis
[94] Azimi et al., 2017	(1) MDA-MB-468 (2) MCF-7	Hypoxia Chamber	N/A	N/A	1% O_2_ for 24 h	Increased ROS production Increased mesenchymal markers (Vimentin, N-cadherin, Snail, Twist, AXL and SERPINE1)	Activating invasion and metastasis
[52] Boidot et al., 2014	20 tumor cell lines	Hypoxia Chamber	24 cycles of 30 min incubation under normoxia and 30 min under hypoxia (1% O_2_)	Cycling hypoxia results in a unique genetic signature that is predictive of clinical outcome in breast cancer patients	1% O_2_ for 24 h	One third of the genes comprising the cycling hypoxic signature and reflects a major difference with the chronic hypoxic signature	Genome instability and mutation
[9] Chen et al., 2018	(1) MMTV-PyMT (2) MCF-7	Hypoxia Chamber	24 h (21% O_2_) followed by 24 h hypoxia (1% O_2_) for 9 days	Increased expression of HIF-1a Increased secretion of tumor growth Promoting cytokine IL-6	1% O_2_ for 9 days	Loss of HIF-1a expression Increased induction of senescent cells	Activating invasion and metastasis
[95] Cooper et al., 2004	(1) MCF-7 (2) ZR-75	Hypoxia Chamber	3 cycles of 64 h hypoxia (0.02 − 0.5% O_2_) and reoxygenation for 8 h	Increased degradation of ER-a protein through altered proteasomal degradation that persisted through reoxygenation	0.02 − 0.5% O_2_ for 24 h	ER-a downregulation	Deregulating cellular energetics
[96] Gutsche et al., 2016	SUM149PT	Hypoxia Chamber	48 h (20.9% O_2_) followed by 24 h hypoxia (0.2% O_2_) for 45 days	Increased expression of pro-metastatic genes (tenascin-C, matrix metalloproteinase 9, COX2)	N/A	N/A	Activating invasion and metastasis
[12] Han et al., 2017	(1) MDA-MB-231 (2) MCF-7	Hypoxia Chamber	20 h (21% O_2_) followed by 4 h hypoxia (1% O_2_)	164 and 242 genes were specifically down- or upregulated only during acute hypoxia	1% O_2_ for 24 h	300 and 623 genes were uniquely down- or upregulated during chronic hypoxic phase	Genome instability and mutation
[11] Jarman et al., 2019	(1) MCF-7 (2) MCF-7-HER2	Hypoxia Chamber	N/A	N/A	0.5% O_2_ for 10 weeks	Overexpression of HER2 leads to upregulation of HIF-2a but not HIF-1a	Sustaining proliferative signaling
[97] Karlenius et al., 2012	MDA-MB-231	Hypoxia Chamber	4 cycles of 10 min hypoxia (0.1% O_2_) and 20 min reoxygenation by placing in 5% CO_2_/95% air	Increase in ROS Increased Trx levels Trx gene promoter is activated in the reoxygenation phase through the action of Nrf-2	0.1% O_2_ for 24 h	Significant decrease in ROS levels No change in Trx levels	Deregulating cellular energetics
[8] Liu et al., 2017	MDA-MB-231	Hypoxia Chamber	12 h (21% O_2_) followed by 12 h hypoxia (1% O_2_) for 5–20 cycles	Significantly increased migration of cells, dependent on the number of cycles Upregulation of HIF-1a and Vimentin	1% O_2_ for 48 h	HIF-1a levels remained low for 36 h	Activating invasion and metastasis
[98] Louie et al., 2010	(1) MDA-MB-231 (2) BCM2	Hypoxia Chamber	1% O_2_ and nutrient deprivation for 7 days, and reoxygenating surviving cells for 1–3 weeks	Stem-like breast cancer subpopulation is expanded responding to reoxygenation	N/A	N/A	Activating invasion and metastasis
[67] Stiehl et al., 2012	MCF-7	Hypoxia Chamber	N/A	N/A	1% O_2_ for 0–72 h	HIF-1a levels peaked at 24–48 h AREG and WISP2 expression was strongly HIF-2a-dependent HIF-2a depletion correlated to a reduction in EGF activation	Sustaining proliferative signaling
[99] Verduzco et al., 2015	MDA-MB-231	Hypoxia Chamber	50 cycles of 16 h hypoxia (0.2% O_2_) followed by 8 h normoxia	Loss of E-cadherin and p53 Increased drug resistance	0.2% O_2_	Few changes in chronic exposed cultures	Activating invasion and metastasis

## Relevance of hypoxia in breast cancer

Hypoxia is generally accepted as an bad prognosis factor in breast cancer patients. Cancer cells largely inhibit protein synthesis in response to hypoxia, and this phenomenon has been termed the oxygen conformance. It details the hierarchy of ATP consuming processes, with DNA, RNA, and protein synthesis being of lesser importance and therefore down-regulated when energy is limited [15]. Hypoxia can lead to inhibition of proliferation and subsequently influence the cell cycle distribution which influences the induction of apoptosis and cell death. The severity of inhibition depends on the degree and duration of hypoxia [16]. Those key experimental parameters required to initiate a response is an essential topic that will be discussed in detail in this review. The hypoxia-induced transcriptional reprogramming within cancer cells has also been shown to allow them to overcome nutrient constraints and support metastatic colonization.

HIF-proteins and tumor hypoxia are known to be closely linked with inducing growth advantage and malignant phenotypes. With respect to metastasis, hypoxia influences two main mechanisms of tumor propagation. The first mechanism is alterations in gene expression which subsequently affect the proteome. The conditions of the hypoxic microenvironment condone hypermutability to DNA damage which is an underlying factor for genomic instability of cancer cells [17]. Higher HIF-1α abundance is also a common feature of breast cancers, due not only to the lack of oxygen but also to mutations in oncogenes or tumor suppressor genes [18]. The second mechanism for alteration is clonal expansion. Hypoxia has been shown to provide selective pressure for expansion of cells with apoptotic resistance, such as p53 mutants, or loss of DNA mismatch repair mechanisms [19, 20].

It is well established in the literature that breast cancer progression is not only regulated by cancer cell signaling but is also influenced by the tumor surrounding environment. The breast microenvironment is comprised mainly of extracellular matrix proteins, endothelial cells, fibroblasts, adipocytes, and immune cells [21]. The breast cancer microenvironment can be characterized at local (within the tumor), regional (within the breast), and distant (metastasis to other organs) levels [22]. Each level is comprised of important regulators of cancer progression such as the growth, structural support (e.g. extracellular matrix), nutrient sources, and physical properties (e.g. pH and oxygen levels) [23]. Induction of a hypoxic environment, and the subsequent activation of HIFs exerts an influence on the microenvironment, which is a decisive step in the uncontrolled growth of the primary tumor. Non-cancer cells within the microenvironment are strongly affected by hypoxia as well. In most cases, non-cancer cell dysregulation supports tumor growth and facilitates metastasis: fibroblasts are modified into tumor conducive cancer associated fibroblasts (CAFs), collagen deposition allows for increased metastasis, and anti-tumor immune functions becomes strongly repressed [24–26].

## Hypoxia and breast cancer treatment resistance

The most detrimental effect of tumor hypoxia is the induction of treatment resistance [3]. Radiotherapy acts through the generation of ROS, which cause irreversible cellular DNA damage and induce apoptosis [27]. Due to the low levels of available oxygen, cancer cells experiencing hypoxia are three-times more resistant to radiation than cells under normoxia [27]. As for chemotherapy, drug treatments showed selective toxicity towards oxygenated cells in comparison to hypoxic cells [28]. The tumor has restricted vasculature, which can manifest as a diffusion barrier between anti-cancer drugs and the tumor itself [29]. The most recent development in cancer treatment is immunotherapy. However, only a minority of patients respond to immunotherapy, in part due to hypoxia [29]. This occurs because the metabolic shift towards upregulated glycolysis results in increased levels of adenosine, which is, amongst other effects, a strong suppressor of T cells [30, 31]. In fact, hypoxia results in an increase in suppressor T cells [32]. Additionally, the adaptive immune system has been shown to be repressed by the action of HIF-1α [31]. Hypoxia has also been shown to downregulate estrogen receptor*-*α expression and function, which could lead to resistance to hormonal therapy as well [33]. Breast cancer is a very heterogenous disease which consists of different subtypes based on the cell of origin within the mammary gland (luminal-like or basal-like) [34]. The basal-like subtype without estrogen receptor, progesterone receptor and HER2 expression is more aggressive and has poor prognosis when compared to the luminal-like subtype [35]. The response of breast cancer cells to hypoxic insult depends on their subtype and therefore, it is another reason to define clear guidelines about the experimental conditions.

## Hypoxia as a parameter in breast cancer research

A summary of key research articles investigated in this review can be found in Table 1. We highlight key findings in studies that compared intermittent hypoxia to acute and chronic hypoxia in various in vitro models of breast cancer. Here we aim to highlight the importance of studying intermittent hypoxia.

### Oxygen concentration during breast cancer progression

**Fig. 2:**
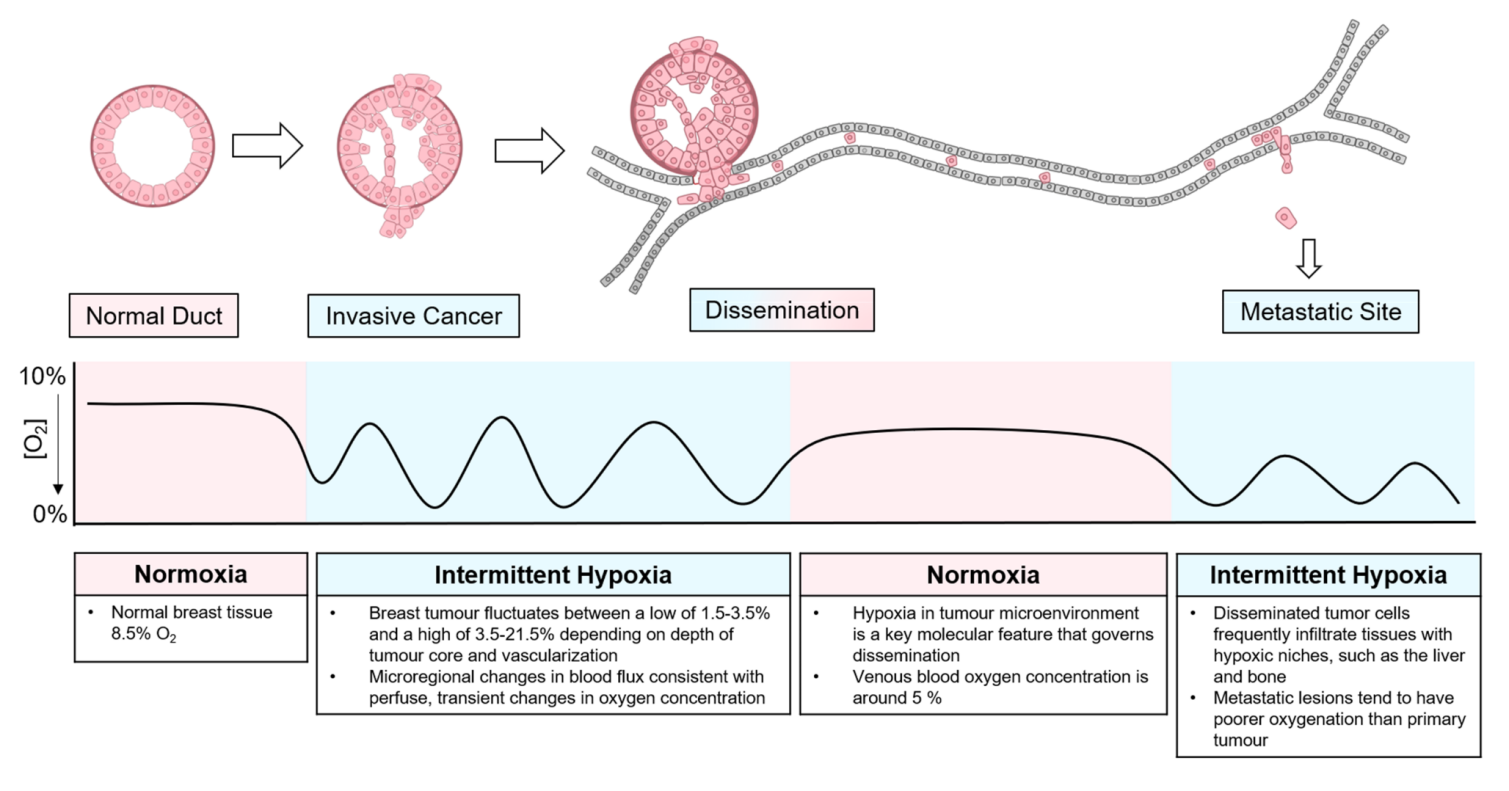
Oxygen concentration during breast cancer progression. As breast cancer progresses from a normal duct tissue, invasive cancer, and dissemination, to metastasis the oxygen concentration of the environment around the cell fluctuates [39, 51, 94–97]

To understand the effects of hypoxia on tumor progression, it is important to conduct the experiments in such a way that they are representative of the pathophysiology of the patient situation. Within a healthy human, oxygen concentrations can vary from 4.6% O_2_ in the brain to 9.5% O_2_ in the renal cortex [36, 37]. However, in cell culture, oxygen concentration is typically maintained at 20% O_2_, indicating that cells are studied under hyperoxic conditions rather than normal physiological conditions [36]. Importantly, the required concentration to induce hypoxia varies among cell types, some cell types are hypoxic at 5% O_2_, while others require less than 1% O_2_ [38]. In human breast tissue, physiological oxygen levels are around 8.5 % O_2_ whereashypoxia in human breast cancer has been determined to be around 1.5% O_2_ [39]. Cells have different responses to low oxygen concentrations. At 1–5% O_2_ the canonical HIF pathway is activated, and other non-canonical pathways can be stimulated to produce the hypoxic response [40]. At around 0.5% O_2_, the cell undergoes reduced mRNA translation, which is the most energetically costly process [40, 41]. At around 0.1% O_2_ and lower, there is reduced respiration and cell cycle arrest [42]. These are important factors to take into consideration when performing experiments that look at cell cycle or mRNA levels in particular. It is also important to realize that the oxygen concentration varies during tumorigenesis. A representation of the stages of hypoxia during breast cancer progression is provided in Fig. 2. At the primary tumor site, both healthy and cancerous cells can experience great fluctuations of oxygen levels depending on the tumor microenvironment. This phase of intra-tumoral intermittent hypoxia is key in driving the molecular features of the metastatic cells. While the circulating cancer cells will get reoxygenated with around 5% of oxygen, the oxygenation status of the metastatic lesions of breast cancers is even poorer than the primary tumors. Additionally local recurrences have also been observed to have a higher hypoxic fraction than the primary tumor [39]. Interestingly, the occurrence of hypoxia in breast cancer does not correlate with the size of the primary tumor and therefore does not correlate with the clinical stage of the disease [39]. Since cancer cells experience different oxygen concentration within the primary tumor, when they enter circulation, and when they colonize a distant organ [43], it is essential to take into consideration what stage is being researched and what the respective oxygen levels for that stage are. Another important factor to take into consideration is that in patients, oxygen is unevenly distributed throughout tissues due to the nature in which oxygen is delivered. Normal oxygen levels in the body flow in a gradient, and as a result areas of hypoxia can also occur in a gradient [44]. Given the presence of flowing oxygen concentration gradients, it can be argued that intermittent hypoxia is most representative of the situation in patients. As seen in Table 1, **1**% O_2_ is most frequently used in the literature, which may be a safe choice as this level of oxygen may not have undesired effects on cell pathophysiology. Nevertheless, it does limit insight into the complex biology of hypoxia in breast cancer.

### Methods of inducing Hypoxia in vitro

As described in Table 1, the two most common methods used to modify oxygen concentration for in vitro studies are (i) having an air-tight chamber providing control over specific gas concentrations, or (ii) inducing a state of hypoxia within the cells biochemically. In the first method, cells are cultured in incubators with the desired concentration of oxygen. There are limitations to this method such as having procedures to sustain hypoxia during times of manual handling of the cultures and taking into account the time required for equilibration of the actual oxygen concentration in the culture [45]. The second method relies on chemicals to initiate signaling events associated with hypoxia, wherein the environment of the cells remains oxygenated. Various chemicals may be used, including- hydroxylase inhibitors, cobalt chloride, nickel chloride, and dimethyloxaloglycine (DMOG), to stabilize HIF-1α giving a phenotype that mimics hypoxia [38, 46, 47]. This method discards the need for complex culture chambers allowing cell culture regular incubators. However, a major limitation is the fact that the scope of the study is narrowed down to effects downstream of the HIF-1α pathway, thus overlooking HIF-1α independent effects of hypoxia [48]. Other drawbacks of chemical induction of hypoxia include the difficulty to study reversibility and intermittent hypoxia and potential toxic effects, such as those associated with cobalt chloride exposure [49, 50]. In patient tumors, the oxygen concentration can vary depending on the location, depth, and size of the tumor [39, 51]. To model this in an experimental setting is complex, and therefore it must be kept in mind that the methods currently used to model hypoxia are not completely representative of the situation in patients. Nevertheless, they can provide important insights into the effects of hypoxia.

### Duration of Hypoxia

As mentioned, the most critical and highly disputed factor when it comes to hypoxia research is the duration of exposure to low oxygen levels. One discrepancy that is seen in Table 1, is the duration of hypoxia required to induce a response. There are three major reasons that can explain these discrepancies. Firstly, for short durations of hypoxia such as from two to thirty minutes [52, 53], it is uncertain at which point the cells are actually under hypoxic conditions. Theoretically, a cell enters hypoxic conditions the moment the incubator door closes. However, in reality it takes several minutes to hours for the oxygen concentration in the medium to asymptotically reach hypoxic levels [54]. Therefore, it is difficult to classify the minimum amount of time required for hypoxia because that can vary between experimental set ups. Secondly, it is not always clear at which point the low oxygen levels stimulate a biological response. Michiels states that within minutes hypoxia induces the activation of HIF proteins, and within hours gene transcription is regulated [55]. The kinetics of HIF-1α in response to reoxygenation was shown to be dependent on the severity of the preceding hypoxic episode [56]. Thirdly, the kinetics of hypoxia induction vary for different methods. For example, it is relatively simple to regulate the hypoxia exposure time using a dedicated chamber whereas this is less straightforward when hypoxia is chemically induced. Ultimately, based on literature we will classify hypoxia as initiating the moment a biological effect occurs.

### Intermittent hypoxia

Intermittent hypoxia is arguably the most important condition to study because it most accurately reflects the situation of oxygenation in tumor tissues. Due to heterogeneous blood supply and dysfunctional vascularization, the oxygen gradient within the tumor is constantly switching between normoxia and hypoxia [57]. Few publications were found comparing intermittent hypoxia to chronic hypoxia in in vitro models of breast cancer. Many studies explore acute or chronic hypoxia but not the effect of subsequent reoxygenation on the cells. Due to its physiological relevance, intermittent hypoxia research is of utmost importance to grow a deeper understanding of the role of hypoxia in breast cancer. Furthermore, it is well known that following treatment with radiation and some forms of chemotherapy, hypoxic tumors will reoxygenate [58]. Therefore, studying effects of intermittent hypoxia would be highly relevant to understand the impact on radiation and chemotherapy.

As seen in Table 1, Alhawarat et al. aimed to compare intermittent and chronic hypoxia on angiogenesis. They found no significant differences between the two groups [59]. However, they produced chronic hypoxic conditions by culturing MCF-7 cells in 1% O_2_ for 72 h, once per week. This means that for the rest of the week the cells were cultured in normoxic conditions, ultimately, leading to intermittent hypoxic conditions. This example highlights the importance of clear definitions of acute, chronic, and intermittent hypoxia.

## Connection between hypoxia exposure regimes and HIF mediated cellular responses

HIFs are the main mediators of cellular responses to hypoxia in the body. HIF is a heterodimer of an α/ β subunit, and each isoform differentially regulates tumor growth [60]. The HIF-1α gene is constitutively transcribed and translated into the HIF-1α protein within the cytosol. Under normoxic conditions, the protein can be ubiquitinated whereby it is marked for degradation by the proteasome. This process is tightly regulated by O_2_ levels through hydroxylation of residues on the oxygen-dependent degradation (ODD) domain. This in turn causes the recruitment of VHL which acts as an E3 ligase to ubiquitinate the protein, marking it for degradation [61]. Under hypoxia, hydroxylation of HIF is inhibited leading to its stabilization and translocation to the nucleus where it forms a complex with HIF-1β. This complex can then bind to the hormone response element (HRE), and with the aid of cofactor p300, initiate transcription of genes involved in the hypoxic response [61]. Besides oxygen levels, HIF-1α can be regulated by growth factor signaling and, in cancer, by activation of oncogenes and loss of tumor suppressor genes [62].

The hypoxic switch is mediated through the binding of HIF-1α and HIF-2α to enhancer elements [63, 64]. HIF-1α and HIF-2α have similar domain structures, are regulated via the same mechanisms, and both bind to HREs and activate HRE- linked reporter genes [44, 65]. In breast cancer cell lines, it has been shown that during hypoxia, HIF-1α expression stabilizes after 4–16 h, then gradually decreases [66], whereas HIF-2α stabilizes after 24 h [67]. Furthermore, HIF-1α and HIF-2α have non-redundant roles, and they produce distinctly different phenotypes [68]. HIF-1α has been shown to be mainly involved in angiogenesis, metabolic reprogramming, invasion, and metastasis [69]. HIF-2α is a key regulator in the promotion of a cancer stem cell phenotype, and stabilizes over a longer time frame [70, 71]. The different roles of HIF-1α and HIF-2α can be explained by the differences in HIF-1α and HIF-2α function and stability. Acute hypoxia is mediated by HIF-1α while HIF-2α may be more dominant in chronic hypoxia [61]. Notably, HIF-1α mRNA has a half-life that is significantly shorter than HIF-2α. Taken together, these results suggest that HIF-1α has a role in acute hypoxia, whereas HIF-2α is active in prolonged, or chronic, responses [72]. Intermittent hypoxia has been shown to induce a response similar to acute hypoxia, with elevated levels of HIF-1α [8]. The level of HIF-2α in response to intermittent hypoxia has yet to be explored in breast cancer cells.

When exposed to hypoxia for a period of 3 days, various luminal breast cancer cell lines showed increased HIF levels, decreased estrogen receptor-α levels, and expressed hallmarks of poor cellular differentiation [73]. Furthermore, it has been shown that this decrease in estrogen receptor-α levels inhibited the growth promoting effects of estradiol, leading to the development of an estrogen-independent phenotype which may explain the acquired resistance to hormonal therapy [33]. In another experiment on ZR-75 cells, this decrease in estrogen receptor-α levels and increase in HIF-1α were shown to be time dependent, the increase in HIF-1α was seen after 3 h of hypoxia exposure, and a decrease in HIF-1α was seen between 6 and 12 h and the levels were almost undetectable after 24 h [74]. When MCF-7 cells were exposed to hypoxia for 16 h a reciprocal relationship was found between HIF-1α and HIF-2α, when HIF-2α was lost, there was a significant increase in HIF-1α-dependent VEGF production, induced by hypoxia [75]. Clearly, HIF-1α stabilization alone does not serve as a surrogate biomarker for the complex hypoxia in breast cancer patients or models. Hypoxia regulated genes controlled by HIF-1α and HIF-2α may be used. However, the same markers may not be used across tumor types. For example, carbonic anhydrase IX (CA9) is used in breast cancer but as it is not expressed in all tumor types would not represent a general biomarker [76].

**Fig. 3:**
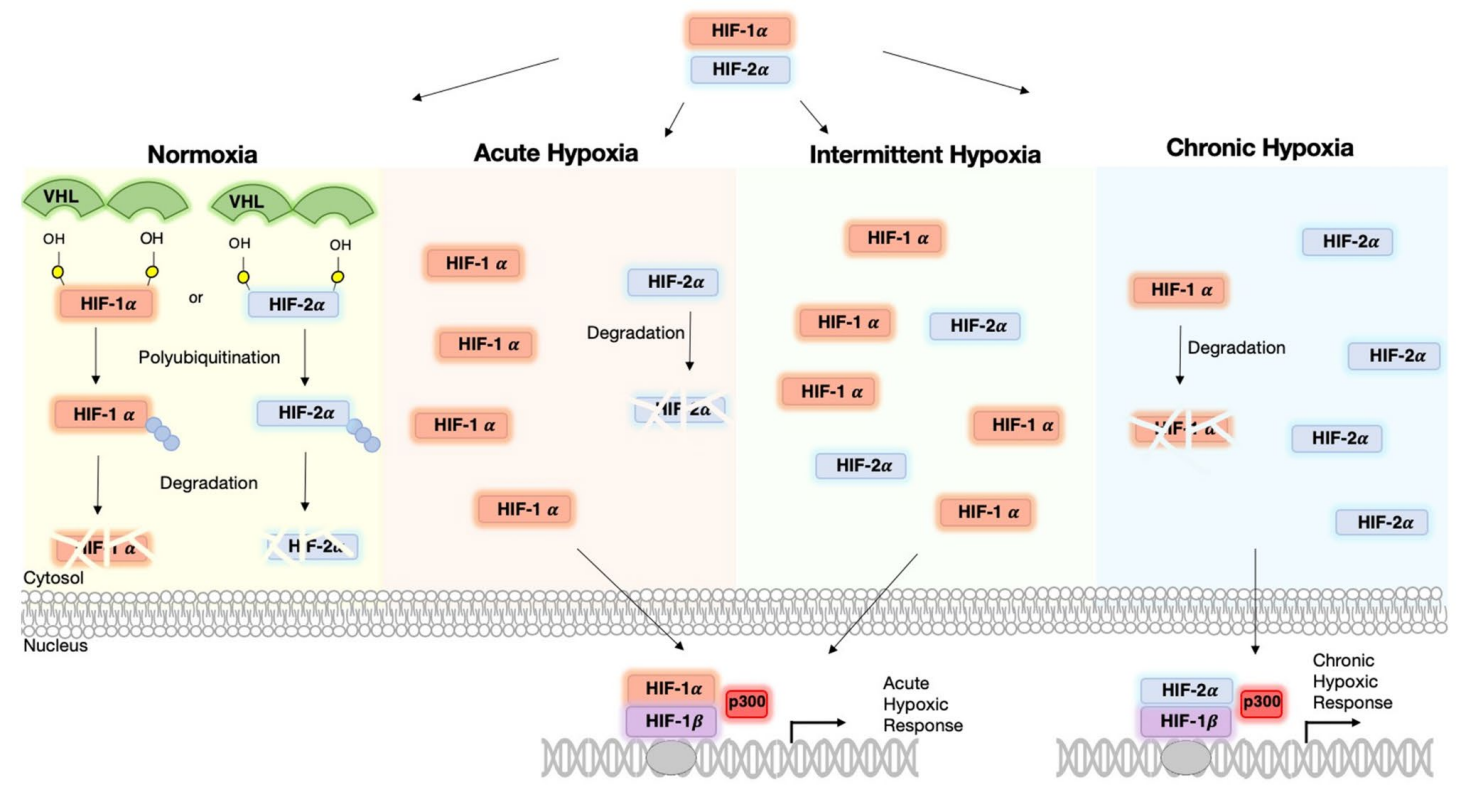
Cellular response in normoxic, acute, intermittent, and chronic hypoxic conditions. HIF proteins are constitutively expressed, but under normal oxygen concentration are quickly ubiquitinated and degraded under the control of VHL. Under acute hypoxia, HIF-1α levels are stabilized and can translocate to the nucleus where they can bind with HIF-1β. With the aid of cofactor p300 gene expression can be induced for the genes involved in acute hypoxic response. Under intermittent hypoxia, HIF-2α levels stabilize alongside HIF-1α levels. Under chronic hypoxia, HIF-1α levels dissipate and HIF-2α levels stabilize. HIF-2α is then the primary cause of gene transcription in chronic hypoxia

HIF-1α and HIF-2α levels vary depending on the duration and degree of hypoxia as summarized in Fig. 3. Both transcription factors mediate distinct responses partially through independent regulation of target genes, but partially through interactions with complexes that contain tumor suppressors and oncoproteins [77]. For example, HIF-1α induced an increase in vascularization, whereas HIF-2α triggered a decrease [78, 79]. Furthermore, acute hypoxia induced secretion of tumor-promoting growth factors and cytokines [80, 81], whereas chronic hypoxia caused deactivation of CAFs [82]. Also studies have shown that HIF-1α but not HIF-2α induces expression of glycolytic genes in multiple cell types [83]. HIF-1α has been shown to activate specifically the expression of LDHA and PDK1, both of which are enzymes that play a critical role in the switch to a primarily glycolytic phenotype [84, 85]. On the contrary, genes that are involved in invasion, including the matrix metalloproteinases (MMP) 2, and 13, and the stem cell factor OCT-3/4, were induced by HIF-2α [61].

**Fig. 4:**
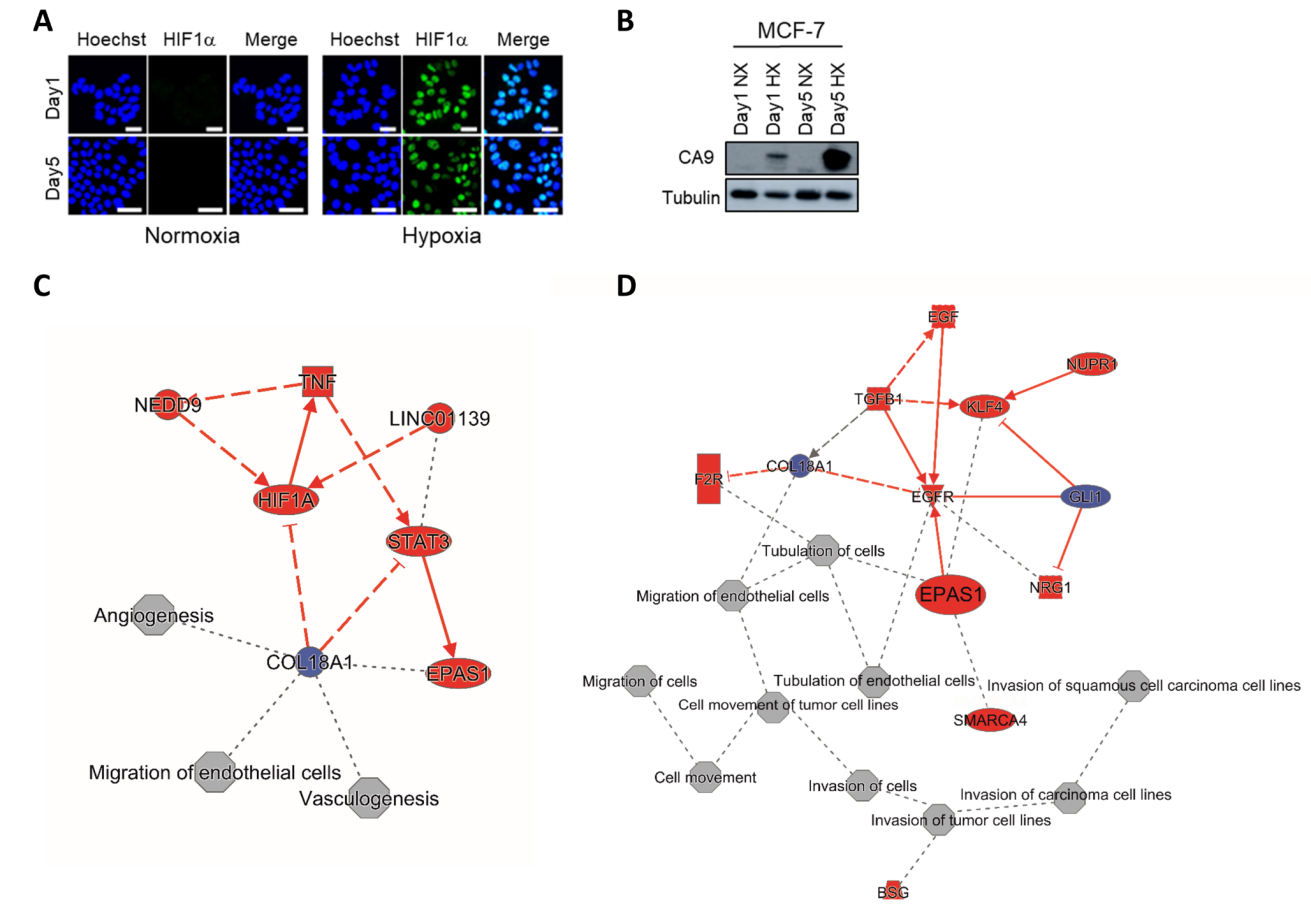
Acute and chronic hypoxia in MCF-7 breast cancer cell line. MCF-7 cells were incubated under normoxia (20% O_2_) or hypoxia (1% O_2_) for 1 day (acute) or 5 days (chronic). Hypoxia biomarkers, HIF1α (A) and CA9 (B), were detected by Immunofluorescence and Western blotting, respectively. Ingenuity Pathway Analysis software was used for analysis of RNAseq data to identify major biological themes for acute (C) and chronic (D) hypoxia in MCF-7 cells. Liu et al., unpublished results

Independent of which HIF binds to the HRE, it ultimately initiates a transcriptional cascade responsible for coordinating a multitude of cellular responses to oxygen availability in many tissues. It controls processes from proliferation, differentiation, metabolism, apoptosis, and the pathophysiology of cancer [86–88]. A schematic of the HIF cascade in response to acute, chronic, and intermittent hypoxia is presented in Fig. 3. The apparent duality between HIF-1α and HIF-2α cascades emphasizes the need for better understanding of hypoxia standards in research. Our own unpublished work points to a shift from HIF-1α to HIF-2α signaling as luminal breast cancer cells are subjected to prolonged hypoxia. I.e., while HIF-1α stabilization and induction of CA9 were observed in MCF7 cells exposed to 1% O_2_ for 24 h or 5 days alike, transcriptome analysis indicated prominent HIF-1α signaling at 24 h and a shift to more prominent HIF-2α (EPAS1) signaling after 5 days hypoxia (Fig. 4). Notably, in addition to the kinetics of hypoxia the impact of varying O_2_ levels is likely to be critically affected by the subtype of breast cancer studied. E.g., basal-like breast cancer cells are more glycolytic than luminal breast cancer cells, which is expected to led to distinct sensitivities to hypoxia [89].

### Connecting hypoxia exposure regimes to hallmarks of cancer

As discussed in this review, there is a discrepancy between what is classified as acute, intermittent, and chronic hypoxia in the current literature. At the same time, we have discussed the critical impact of hypoxia kinetics on the balance between HIF-1α and HIF-2α signaling and, consequently, on affected cellular programs. Hence, studying the impact of hypoxia on described hallmarks of cancer [90] will have to take these considerations into account. Distinct connections between acute, chronic, and intermittent hypoxia in in vitro breast cancer models to sustaining proliferative signaling, activating invasion and metastasis, genome instability and mutation, deregulating cellular energetics, and inducing angiogenesis are depicted in Fig. 1.

For genome instability, HIF-1α has been shown to induce microRNAs which suppress DNA repair mechanisms and can lead to genetic instability [91]. On the other hand, HIF-2α has been shown not to have these same effects due to Thr-324 phosphorylation in the PAS-b domain, which prevents it from suppressing DNA repair genes [92]. Consequently, conclusions drawn from experiments using acute/ intermittent hypoxia (associated with HIF-1α signaling) may be very different from conclusions drawn from experiments using chronic hypoxia (which drives HIF-2α signaling). Likewise, studies investigating the impact of hypoxia on aspects of angiogenesis will critically depend on the O_2_ exposure kinetics. HIF-1α promotes cell proliferation and migration in early angiogenesis, whereas HIF-2α plays a role in remodeling and maturation of the microvasculature controlling vascular morphogenesis and assembly [93]. Based on our own unpublished work, acute hypoxia in luminal breast cancer cell impacts on angiogenesis whereas chronic hypoxia affects processes associated with invasion and metastasis **(**Fig. 4**)**.

## Conclusion & future direction

The aim of this review was to highlight the different strategies being applied to hypoxia research within the field of breast cancer. The lack of definition of time frames, oxygen concentration, and biological effects in in vitro models of acute, chronic or intermittent hypoxia hamper clear conclusions that can be drawn from research in this field. As a minimum guideline for a better definition of acute, chronic, and intermittent hypoxia we propose: (i) acute hypoxia is when the cells are exposed for no more than 24 h to an environment with 1% O_2_ or less. This time frame has been chosen based on the literature because it is the period in which HIF-1α is stabilized and most active; (ii) chronic hypoxia is when the cells are exposed for more than 48 h to an environment with 1% O_2_ or less. This time frame has been selected based on the literature as the timing for HIF-2α activation; (iii) intermittent hypoxia is when the cells are exposed to at least two rounds of hypoxia (1% O_2_ or less) separated by at least one period of reoxygenation by exposure to normoxia (8.5% O_2_ or higher). Studies investigating the changes in signaling, gene expression, and metabolism using in vitro breast cancer models under such defined conditions should serve to build a stronger foundation within the field and provide better insight into the role of hypoxia role in breast cancer progression and therapy resistance.

## Electronic supplementary material

Below is the link to the electronic supplementary material.

Supplementary Material 1
