# Supplementary material for: Acute vs. chronic vs. intermittent hypoxia in breast Cancer: a review on its application in in vitro research
Source: Mol Biol Rep. 2022 Sep 3;49(11):10961–73. doi: 10.1007/s11033-022-07802-6 (PMC9618509; doi:10.1007/s11033-022-07802-6)
Supplement: Supplementary file 1 — Supplementary Material 1 [file 11033_2022_7802_MOESM1_ESM.docx]

Reference

Old ones are labelled yellow.

1 Muz B, La Puente P de, Azab F et al. (2015) The role of hypoxia in cancer progression, angiogenesis, metastasis, and resistance to therapy. Hypoxia (Auckl) 3:83–92. <https://doi.org/10.2147/HP.S93413>

1 Sørensen BS, Horsman MR (2020) Tumor Hypoxia: Impact on Radiation Therapy and Molecular Pathways. Front Oncol 10:562. https://doi.org/10.3389/fonc.2020.00562

3 Semenza GL (2016) The hypoxic tumor microenvironment: A driving force for breast cancer progression. Biochim Biophys Acta 1863:382–391. <https://doi.org/10.1016/j.bbamcr.2015.05.036>

3 Zhang Y, Zhang H, Wang M et al. (2021) Hypoxia in Breast Cancer-Scientific Translation to Therapeutic and Diagnostic Clinical Applications. Front Oncol 11:652266. https://doi.org/10.3389/fonc.2021.652266

4 Brizel DM, Scully SP, Harrelson JM et al. (1996) Tumor oxygenation predicts for the likelihood of distant metastases in human soft tissue sarcoma. Cancer Res 56:941–943

4 Tutzauer J, Sjöström M, Holmberg E et al. (2022) Breast cancer hypoxia in relation to prognosis and benefit from radiotherapy after breast-conserving surgery in a large, randomised trial with long-term follow-up. Br J Cancer 126:1145–1156. https://doi.org/10.1038/s41416-021-01630-4

5 Mees G, Dierckx R, Vangestel C et al. (2009) Molecular imaging of hypoxia with radiolabelled agents. Eur J Nucl Med Mol Imaging 36:1674–1686. <https://doi.org/10.1007/s00259-009-1195-9>

5 Cui J, Jiang H (2019) Prediction of postoperative survival of triple-negative breast cancer based on nomogram model combined with expression of HIF-1α and c-myc. Medicine (Baltimore) 98:e17370. <https://doi.org/10.1097/MD.0000000000017370>

6 Michiels C, Tellier C, Feron O (2016) Cycling hypoxia: A key feature of the tumor microenvironment. Biochim Biophys Acta 1866:76–86. <https://doi.org/10.1016/j.bbcan.2016.06.004>

6 Bader SB, Dewhirst MW, Hammond EM (2020) Cyclic Hypoxia: An Update on Its Characteristics, Methods to Measure It and Biological Implications in Cancer. Cancers (Basel) 13. https://doi.org/10.3390/cancers13010023

10 Matschke J, Riffkin H, Klein D et al. (2016) Targeted Inhibition of Glutamine-Dependent Glutathione Metabolism Overcomes Death Resistance Induced by Chronic Cycling Hypoxia. Antioxid Redox Signal 25:89–107. <https://doi.org/10.1089/ars.2015.6589>

10 Hansel C, Hlouschek J, Xiang K et al. (2021) Adaptation to Chronic-Cycling Hypoxia Renders Cancer Cells Resistant to MTH1-Inhibitor Treatment Which Can Be Counteracted by Glutathione Depletion. Cells 10. <https://doi.org/10.3390/cells10113040>

14 Hanahan D, Weinberg RA (2011) Hallmarks of cancer: the next generation. Cell 144:646–674. <https://doi.org/10.1016/j.cell.2011.02.013>

14 Hanahan D (2022) Hallmarks of Cancer: New Dimensions. Cancer Discov 12:31–46. <https://doi.org/10.1158/2159-8290.CD-21-1059>

15 Buttgereit F, Brand MD (1995) A hierarchy of ATP-consuming processes in mammalian cells. Biochem J 312 (Pt 1):163–167. <https://doi.org/10.1042/bj3120163>

15 Yang G, Shi R, Zhang Q (2020) Hypoxia and Oxygen-Sensing Signaling in Gene Regulation and Cancer Progression. Int J Mol Sci 21. https://doi.org/10.3390/ijms21218162

18 Semenza GL (2003) Targeting HIF-1 for cancer therapy. Nat Rev Cancer 3:721–732. <https://doi.org/10.1038/nrc1187>

18 Sharma A, Sinha S, Shrivastava N (2022) Therapeutic Targeting Hypoxia-Inducible Factor (HIF-1) in Cancer: Cutting Gordian Knot of Cancer Cell Metabolism. Front Genet 13:849040. https://doi.org/10.3389/fgene.2022.849040

19 Graeber TG, Osmanian C, Jacks T et al. (1996) Hypoxia-mediated selection of cells with diminished apoptotic potential in solid tumours. Nature 379:88–91.

[https://doi.org/10.1038/379088a0](https://doi.org/10.1038/379088a0 19)

[19](https://doi.org/10.1038/379088a0 19) Tang M, Bolderson E, O'Byrne KJ et al. (2021) Tumor Hypoxia Drives Genomic Instability. Front Cell Dev Biol 9:626229. https://doi.org/10.3389/fcell.2021.626229

20 Kondo A, Safaei R, Mishima M et al. (2001) Hypoxia-induced enrichment and mutagenesis of cells that have lost DNA mismatch repair. Cancer Res 61:7603–7607

20 Bhandari V, Li CH, Bristow RG et al. (2020) Divergent mutational processes distinguish hypoxic and normoxic tumours. Nat Commun 11:737. https://doi.org/10.1186/s12859-019-2610-2

21 Place AE, Jin Huh S, Polyak K (2011) The microenvironment in breast cancer progression: biology and implications for treatment. Breast Cancer Res 13:227. <https://doi.org/10.1186/bcr2912>

21 Fico F, Santamaria-Martínez A (2020) The Tumor Microenvironment as a Driving Force of Breast Cancer Stem Cell Plasticity. Cancers (Basel) 12. https://doi.org/10.3390/cancers12123863

24 Bergers G, Benjamin LE (2003) Tumorigenesis and the angiogenic switch. Nat Rev Cancer 3:401–410. <https://doi.org/10.1038/nrc1093>

24 Piper M, Mueller AC, Karam SD (2020) The interplay between cancer associated fibroblasts and immune cells in the context of radiation therapy. Mol Carcinog 59:754–765. <https://doi.org/10.1002/mc.23205>

25 Kauppila S, Stenbäck F, Risteli J et al. (1998) Aberrant type I and type III collagen gene expression in human breast cancer in vivo. J Pathol 186:262–268. [https://doi.org/10.1002/(SICI)1096-9896(1998110)186:3<262:AID-PATH191>3.0.CO;2-3](https://doi.org/10.1002/(SICI)1096-9896(1998110)186:3%3c262:AID-PATH191%3e3.0.CO;2-3)

25 Nissen NI, Karsdal M, Willumsen N (2019) Collagens and Cancer associated fibroblasts in the reactive stroma and its relation to Cancer biology. J Exp Clin Cancer Res 38:115. https://doi.org/10.1186/s13046-019-1110-6

26 Sitkovsky M, Lukashev D (2005) Regulation of immune cells by local-tissue oxygen tension: HIF1 alpha and adenosine receptors. Nat Rev Immunol 5:712–721. <https://doi.org/10.1038/nri1685>

26 Steingold JM, Hatfield SM (2020) Targeting Hypoxia-A2A Adenosinergic Immunosuppression of Antitumor T Cells During Cancer Immunotherapy. Front Immunol 11:570041. https://doi.org/10.3389/fimmu.2020.570041

27 Teicher BA, Lazo JS, Sartorelli AC (1981) Classification of antineoplastic agents by their selective toxicities toward oxygenated and hypoxic tumor cells. Cancer Res 41:73–81

27 Rockwell S, Dobrucki IT, Kim EY et al. (2009) Hypoxia and radiation therapy: past history, ongoing research, and future promise. Curr Mol Med 9:442–458. https://doi.org/10.2174/156652409788167087

31 Facciabene A, Peng X, Hagemann IS et al. (2011) Tumour hypoxia promotes tolerance and angiogenesis via CCL28 and T(reg) cells. Nature 475:226–230. <https://doi.org/10.1038/nature10169>

31 Leone RD, Horton MR, Powell JD (2015) Something in the air: hyperoxic conditioning of the tumor microenvironment for enhanced immunotherapy. Cancer Cell 27:435–436. https://doi.org/10.1016/j.ccell.2015.03.014

33 Müller M, Padberg W, Schindler E et al. (1998) Renocortical tissue oxygen pressure measurements in patients undergoing living donor kidney transplantation. Anesth Analg 87:474–476. <https://doi.org/10.1097/00000539-199808000-00045>

33 Rani A, Stebbing J, Giamas G et al. (2019) Endocrine Resistance in Hormone Receptor Positive Breast Cancer-From Mechanism to Therapy. Front Endocrinol (Lausanne) 10:245. <https://doi.org/10.3389/fendo.2019.00245>

34. Bertucci F, Finetti P, Goncalves A et al. (2020) The therapeutic response of ER+/HER2- breast cancers differs according to the molecular Basal or Luminal subtype. NPJ Breast Cancer 6:8. https://doi.org/10.1038/s41523-020-0151-5

35 Rockwell S, Dobrucki IT, Kim EY et al. (2009) Hypoxia and radiation therapy: past history, ongoing research, and future promise. Curr Mol Med 9:442–458. <https://doi.org/10.2174/156652409788167087>

35 Fan M, Chen J, Gao J et al. (2020) Triggering a switch from basal- to luminal-like breast cancer subtype by the small-molecule diptoindonesin G via induction of GABARAPL1. Cell Death Dis 11:635. https://doi.org/10.1038/s41419-020-02878-z

44 Piret J-P, Mottet D, Raes M et al. (2002) CoCl2, a chemical inducer of hypoxia-inducible factor-1, and hypoxia reduce apoptotic cell death in hepatoma cell line HepG2. Ann N Y Acad Sci 973:443–447. <https://doi.org/10.1111/j.1749-6632.2002.tb04680.x>

44 Wenger RH (2002) Cellular adaptation to hypoxia: O2-sensing protein hydroxylases, hypoxia-inducible transcription factors, and O2-regulated gene expression. FASEB J 16:1151–1162. <https://doi.org/10.1096/fj.01-0944rev>

55 Kimura H, Braun RD, Ong ET et al. (1996) Fluctuations in red cell flux in tumor microvessels can lead to transient hypoxia and reoxygenation in tumor parenchyma. Cancer Res 56:5522–5528

55 Michiels C (2004) Physiological and pathological responses to hypoxia. Am J Pathol 164:1875–1882. <https://doi.org/10.1016/S0002-9440(10)63747-9>

61 Semenza GL, Wang GL (1992) A nuclear factor induced by hypoxia via de novo protein synthesis binds to the human erythropoietin gene enhancer at a site required for transcriptional activation. Mol Cell Biol 12:5447–5454. <https://doi.org/10.1128/mcb.12.12.5447-5454.1992>

61 Koh MY, Lemos R, Liu X et al. (2011) The hypoxia-associated factor switches cells from HIF-1α- to HIF-2α-dependent signaling promoting stem cell characteristics, aggressive tumor growth and invasion. Cancer Res 71:4015–4027. https://doi.org/10.1158/0008-5472.CAN-10-4142

62 Tian H, McKnight SL, Russell DW (1997) Endothelial PAS domain protein 1 (EPAS1), a transcription factor selectively expressed in endothelial cells. Genes Dev 11:72–82. <https://doi.org/10.1101/gad.11.1.72>

62 Masoud GN, Li W (2015) HIF-1α pathway: role, regulation and intervention for cancer therapy. Acta Pharm Sin B 5:378–389. <https://doi.org/10.1016/j.apsb.2015.05.007>

63 Wiesener MS, Turley H, Allen WE et al. (1998) Induction of endothelial PAS domain protein-1 by hypoxia: characterization and comparison with hypoxia-inducible factor-1alpha. Blood 92:2260–226

63 Albadari N, Deng S, Li W (2019) The transcriptional factors HIF-1 and HIF-2 and their novel inhibitors in cancer therapy. Expert Opin Drug Discov 14:667–682. https://doi.org/10.1080/17460441.2019.1613370

74 Le Q-T, Courter D (2008) Clinical biomarkers for hypoxia targeting. Cancer Metastasis Rev 27:351–362. <https://doi.org/10.1007/s10555-008-9144-9>.

74 Stoner M, Saville B, Wormke M et al. (2002) Hypoxia induces proteasome-dependent degradation of estrogen receptor alpha in ZR-75 breast cancer cells. Mol Endocrinol 16:2231–2242. <https://doi.org/10.1210/me.2001-0347>

77 Tang N, Wang L, Esko J et al. (2004) Loss of HIF-1alpha in endothelial cells disrupts a hypoxia-driven VEGF autocrine loop necessary for tumorigenesis. Cancer Cell 6:485–495. <https://doi.org/10.1016/j.ccr.2004.09.026>

77 Keith B, Johnson RS, Simon MC (2011) HIF1α and HIF2α: sibling rivalry in hypoxic tumour growth and progression. Nat Rev Cancer 12:9–22. https://doi.org/10.1038/nrc3183

82 Kim J-w, Tchernyshyov I, Semenza GL et al. (2006) HIF-1-mediated expression of pyruvate dehydrogenase kinase: a metabolic switch required for cellular adaptation to hypoxia. Cell Metab 3:177–185. <https://doi.org/10.1016/j.cmet.2006.02.002>

82 Madsen CD, Pedersen JT, Venning FA et al. (2015) Hypoxia and loss of PHD2 inactivate stromal fibroblasts to decrease tumour stiffness and metastasis. EMBO Rep 16:1394–1408. <https://doi.org/10.15252/embr.201540107>

83 Semenza GL, Jiang BH, Leung SW et al. (1996) Hypoxia response elements in the aldolase A, enolase 1, and lactate dehydrogenase A gene promoters contain essential binding sites for hypoxia-inducible factor 1. J Biol Chem 271:32529–32537. <https://doi.org/10.1074/jbc.271.51.32529>

83 Hu C-J, Wang L-Y, Chodosh LA et al. (2003) Differential roles of hypoxia-inducible factor 1alpha (HIF-1alpha) and HIF-2alpha in hypoxic gene regulation. Mol Cell Biol 23:9361–9374. https://doi.org/10.1128/MCB.23.24.9361-9374.2003

98 Hill SA, Pigott KH, Saunders MI et al. (1996) Microregional blood flow in murine and human tumours assessed using laser Doppler microprobes. Br J Cancer Suppl 27:S260-3

99 Pigott KH, Hill SA, Chaplin DJ et al. (1996) Microregional fluctuations in perfusion within human tumours detected using laser Doppler flowmetry. Radiotherapy and Oncology 40:45–50. https://doi.org/10.1016/0167-8140(96)01730-6
